# Cost-effective strategies for CAR-T cell therapy manufacturing

**DOI:** 10.1016/j.omton.2025.200980

**Published:** 2025-04-03

**Authors:** Luiza Abdo, Leonardo Ribeiro Batista-Silva, Martín Hernán Bonamino

**Affiliations:** 1 Cell and Gene Therapy Program, Research Coordination, National Cancer Institute (INCA), Rio de Janeiro 20231-050, Brazil; 2 Vice-Presidency of Research and Biological Collections (VPPCB), Oswaldo Cruz Foundation (FIOCRUZ), Rio de Janeiro 21040-900, Brazil

**Keywords:** MT: Regular Issue, CAR-T cell, cost-effective, immunotherapy, manufacturing, B cell tumor

## Abstract

CAR-T cell therapy has revolutionized cancer treatment, with approvals for conditions like acute B-leukemia, large B cell lymphoma (LBCL), follicular lymphoma (FL), mantle cell lymphoma (MCL), and multiple myeloma. However, its high costs limit accessibility. Key factors driving these costs include the need for personalized, autologous treatments, transportation to specialized facilities, reliance on viral vectors requiring advanced laboratories, and lengthy cell expansion processes. To address these challenges, alternative strategies aim to simplify and reduce production complexity. Non-viral vectors, such as Sleeping Beauty, piggyBac, and CRISPR, delivered via nanoparticles or electroporation, present promising solutions. These methods could streamline manufacturing, eliminate the need for viral vectors, and reduce associated costs. Furthermore, shortening cell expansion periods and optimizing protocols could significantly accelerate production. An emerging approach involves using genetically edited T cells from healthy donors to create universal CAR-T products capable of treating multiple patients. Finally, decentralized point-of-care (POC) manufacturing of CAR-T cells minimize logistical expenses, eliminating the need for complex infrastructure, and enabling localized production closer to patients. This innovative strategy holds potential for broadening access and reducing costs, representing a step toward democratizing CAR-T therapy. Combined, these advances could make this groundbreaking treatment more feasible for healthcare systems worldwide.

## Graphical abstract

**Figure:**
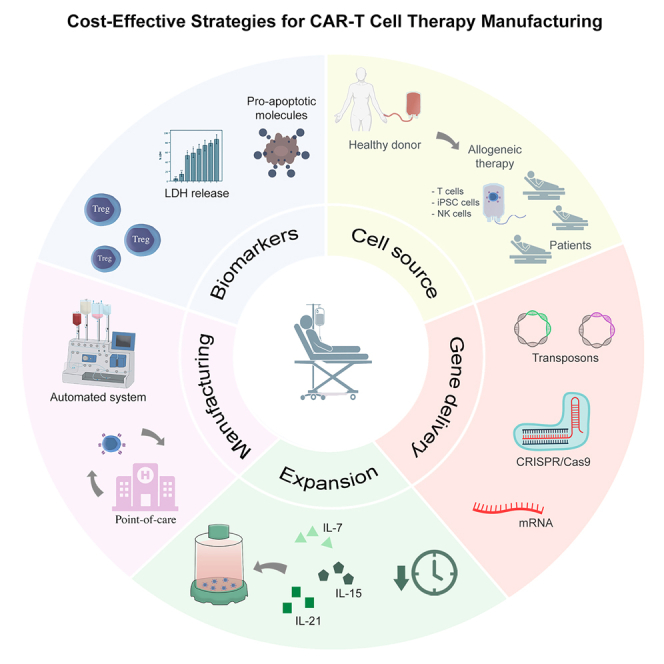

## Teaser

CAR-T therapy has transformed cancer treatment, but high costs limit accessibility. Alternatives such as non-viral vectors, shorter expansion times, universal CAR-T cells, and decentralized manufacturing can help reduce costs. This review describes these strategies to consolidate information that may contribute to the democratization of CAR-T cell therapy.

## Introduction

Harnessing the patient’s own immune system to recognize and eliminate tumors has emerged as a major cancer treatment modality, representing a significant advancement in cancer immunotherapy. One strategy to achieve this goal involves the genetic modification of autologous T cells to express chimeric antigen receptors (CARs), enabling them to recognize specific molecules on target cells for elimination. Since its first approval by the Food and Drug Administration (FDA), the use of this therapy has increased, offering a new perspective on cancer treatment for various types of refractory hematological malignancies that were previously considered incurable. As of February 2025, the FDA has approved seven CAR-T cell therapies: four anti-CD19 therapies for the treatment of B cell-derived leukemia and/or lymphoma (axicabtagene ciloleucel, brexucabtagene autoleucel, lisocabtagene maraleucel, tisagenlecleucel, and obecabtagen autolocel), and two anti-BCMA therapies for the treatment of multiple myeloma (ciltacabtagene autoleucel and idecabtagene vicleucel). Recent long-term follow-up data from 12 studies of CD19-targeted CAR-T therapy products in B-ALL patients (median follow-up 1–4.8 years) confirm excellent initial complete remission (CR) rates of 62%–86%, with most achieving deep MRD-negative remissions. For commercially available products, tisagenlecleucel demonstrated a 69% CR rate and median EFS of 5.6 months at 13 months follow-up, while brexucabtagene autoleucel showed a 69% CR rate and 7-month median relapse-free survival at 22 months. In pediatric B-ALL, tisagenlecleucel achieved an 82% CR rate and 24-month median EFS in the ELIANA trial. For BCMA-targeted therapies, idecabtagene vicleucel reported a 33% CR rate and 19-month median response duration, while ciltacabtagene autoleucel achieved an 83% CR rate, 55% PFS at 27 months, and a non-estimable median response duration. These findings underscore the efficacy and durability of CAR-T therapies while highlighting the need for extended follow-up to optimize outcomes.^1^

Over the past decades, advancements in understanding T cell functionality have enabled the development of CAR molecules. These artificial constructs combine components derived from antibodies, intracellular TCR signaling domains, and co-stimulatory molecules, designed to activate genetically modified T lymphocytes. CARs aim to replicate, at least partially, the canonical signaling processes of T cells that occur during peptide-MHC complex recognition. Typically, a CAR molecule consists of an extracellular antigen recognition domain composed of a single-chain variable fragment (scFv) derived from a monoclonal antibody. This scFv contains variable light (VL) and variable heavy (VH) chains connected into a single polypeptide chain. The scFv is linked to a flexible hinge region that provides structural adaptability, which is further connected to a transmembrane domain. Another alternative extracellular domain that has been gaining attention is the use of nanobodies instead of scFv. Nanobodies are single-domain antibodies composed only of the domains of heavy-chain-only antibodies. This makes a smaller antigen-recognition unit, allowing it to bind to epitopes closer to the target cell membrane. The transmembrane domain, often derived from CD8α, CD28, or IgG4, ensures stability and efficient signal transmission across the cell membrane. The intracellular signaling region of the CAR includes the zeta chain of the CD3 complex (CD3ζ), responsible for initiating the primary activation signal (signal 1). Additionally, a co-stimulatory domain, typically derived from molecules such as CD28 or 4-1BB, provides the secondary signal (signal 2) needed to enhance T cell proliferation, persistence, and function. Although this basic structure is widely adopted, CARs are highly versatile. Numerous combinations of antigen recognition domains, hinge regions, transmembrane segments, and intracellular signaling elements have been developed to optimize their performance for diverse therapeutic applications.^2^^,^^3^ Recently, the approved obecabtagen autolocel (Aucatzyl) was designed with a low-affinity scFv (>40-fold lower compared to FMC63), which enables faster dissociation from the CD19 target compared to traditional CARs. This rapid release reduces the risk of severe side effects such as CRS and ICANS. Furthermore, the transient interaction helps prevent CAR-T cell exhaustion, promoting their persistence in the body. The concept of the “fast-off” effect is based on the ligand-binding dynamics of the CAR-T cell scFv, allowing for maintained therapeutic efficacy while offering an improved safety profile for patients.^4^

The long-term outcomes for patients with incurable cancer treated with CAR-T cell therapy are promising. In B cell acute lymphoblastic leukemia (B-ALL) patients, complete response rates range from 62% to 86% in studies with a follow-up of at least 13 months. For patients with B cell lymphoma, the overall response rate (ORR) varies between 44% and 91% in studies with a minimum follow-up of 34 months. In relapsed and/or refractory multiple myeloma patients treated with BCMA-targeted CAR-T cells, response rates range from 73% to 100%, with at least 13 months of monitoring.^1^

It is important to emphasize that the high success rates of CAR-T cell therapy are observed in patients who have failed conventional therapies, further underscoring the effectiveness of this approach. However, despite the high response rates achieved in most treated patients, one of the main barriers to accessing this therapy is the cost of commercial CAR-T cell products. The price reflects the substantial expenses associated with production, logistics, quality control, and profit margins. When combined with the costs of contracting manufacturers, patient management, and hospital fees, the total expense for a single treatment can reach up to $2 million per individual in the United States.^5^ In fact, this is the major obstacle to the democratization of this immunotherapy.

Given this context, it is essential to develop new approaches to make CAR-T cell therapy more accessible. The use of different gene editing tools, optimization of the production process, reduction of CAR-T cell expansion time and decentralization of manufacturing could be interesting approaches to achieve cost reduction. We herein gather initiatives with potential to help reduce the costs of this disruptive immunotherapy. Here, we will describe possible alternatives which, if put into practice, could bring several improvements to patients who can benefit from CAR-T cell therapy.

## Current manufacturing

Manufacturing of CAR T cells is based on patient-derived lymphocytes undergoing genetic modification, activation, and *in vitro* expansion to get hundreds of millions of CAR-T cells before reinfusion. Currently, the manufacturing process comprises several sequential steps. It begins at the healthcare facility with leukapheresis and the separation of patient peripheral blood mononuclear cells (PBMCs) using a density gradient method, followed by centrifugation to eliminate red blood cells and platelets. Usually, the isolation of PBMCs from patient’s whole blood is mostly collected through leukapheresis procedures at accredited hospital units where the patient is undergoing treatment. After patient’s PBMC collection, the leukapheresis can be cryopreserved before shipping it from the healthcare facility to a central cell therapy manufacturing laboratory. Currently, the pharmaceutical industries with commercially approved products have facilities only in North America, Asia, and European Union to supply the markets around the world. Once the product arrives at the central manufacturing laboratory, the leukapheresis is submitted to T lymphocytes enrichment employing a magnetic column with anti-CD3 or anti-CD4/CD8 through positive selection. Enriched T cells are cultured and expanded for around one week in the presence of anti-CD3/CD28 stimulating reagents, being transduced with viral vectors to express the CAR transgene in media supplemented with cytokines (usually IL-2 or combinations of IL-7 and IL-15). The CAR transgenes most commonly target a specific antigen such as CD19 or BCMA in commercial products. Raising CAR targets targeting solid tumors such as HER2, GD2, EGFRvIII among others are currently under evaluation in clinical trials.^6^^,^^7^^,^^8^

The current CAR-T cell manufacturing process for the approved products use retroviral or lentiviral vectors for the genetic modification of T lymphocytes.^9^^,^^10^ In fact, the commercially available products have underscored the substantial efficacy of CAR-T cell therapy targeting CD19 in treating relapsed/refractory B cell lymphomas and adults with relapsed/refractory DLBCL.^11^ There are many advantages of using viral vectors such as high transgene insertion efficiency, excellent cellular viability after gene delivery, elevated expression of the CAR molecule, and limited genotoxicity. But, on the other hand, the production of viral batches itself significantly increases the cost of this therapy. Currently, each viral batch for a single patient can cost more than $16,000,^12^ making non-viral delivery methods more attractive. Those viral vectors can be derived from various types of viruses, including adenoviruses, adeno-associated viruses, and lentiviruses. Lentiviral and gamma-retroviral vectors are commonly used in gene therapy to deliver specific genes into cells, particularly for conditions where integrative expression-introduced gene is required.^13^^,^^14^^,^^15^ Although these vectors are generally assumed to be safe, recent reports of T cell transformation following gene modification to generate CAR-T cells have raised concerns about insertional mutagenesis in the long term. The CAR-T community is carefully evaluating the rate of T cell transformation related to CAR therapy.^16^ However, a study recently published by Jadlowsky and colleagues offers critical insights into the genomic safety profile of CAR-T cell therapies by conducting a comprehensive analysis of vector integration sites across 176 patients. The evaluation of integration vector profiles revealed no pathological insertions directly associated with the development of secondary neoplasms, supporting the overall safety of these therapeutic interventions. The study identified instances of vector integration in proximity to specific genomic loci, including tumor suppressor genes, which correlated with modest clonal expansion and prolonged persistence of the engineered T cell populations. These observations suggest that, while the risk of insertional mutagenesis leading to oncogenic transformation remains low, the spatial distribution of integration events near critical regulatory regions may influence clonal dynamics and T cell longevity. These findings highlight the necessity for ongoing genomic surveillance and extended follow-up studies to further elucidate the long-term implications of vector integration patterns and to refine the safety parameters of CAR-T cell-based immunotherapies.^17^

After the expansion of modified CAR-T cells to reach the required cell numbers for treatment, the cells are formulated and/or cryopreserved prior to reinfusion. The cell products then undergo several quality control assays and must meet release criteria, including (1) sterility, (2) cell identity, (3) minimal percentage of cells expressing the CAR molecule, (4) absence of replication-competent vectors, (5) viability, and (6) DNA-integrated vector copy number evaluation. It is important to note that CAR-T cell manufacturing currently requires good manufacturing practice (GMP) facilities, which consist of complex infrastructure and systems to ensure compliance with GMP regulations. At the end of the CAR-T cell manufacturing process and cryopreservation, the final product is shipped back to the healthcare unit, where the cells will be infused into the patient. The complete vein-to-vein process can take up to 30 days and, in many cases, longer. This lengthy manufacturing period can be challenging for patients with rapidly progressive disease.^18^ Additionally, it is often necessary to transport the product over long distances from the manufacturing center to the patient handling facility to receive the CAR-T cells under ideal conditions. It is important to note that the cryopreservation process can potentially affect both the viability and efficacy of the T cells.^19^ Once the product is cleared and available, the patient can be conditioned to receive the infusion of the CAR-T product.

**Figure 1:**
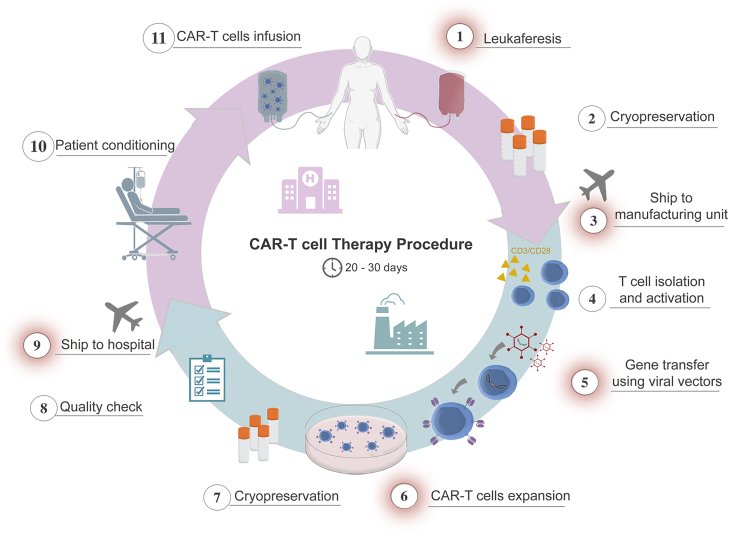
CAR-T cell product manufacturing involves several steps, which increase the complexity and cost of this therapy After a patient is indicated for this therapy, the procedure is generally divided into two main phases: the steps carried out in the hospital (shown in purple) and the steps carried out in the manufacturing unit (shown in blue). In the hospital, the patient undergoes leukapheresis and cryopreservation of the cells (if required). The cells are then transported to the production center, which may be in a different country. At the manufacturing unit, T cells are isolated, activated, modified to express the CAR through viral transduction, expanded for several days, frozen, and subjected to quality control. The cells are then shipped back to the hospital. Finally, the patient undergoes conditioning and receives the CAR-T cells. This procedure can take up to one month, depending on the origin of the cells. Efforts to simplify and optimize this production process are currently underway, with major points for improvement highlighted in red in the figure.

In the diagram mentioned further, we illustrate the current standard process for manufacturing CAR-T cells, highlighting the primary obstacles to their improvement. We discuss herein potential process optimizations, including refining the leukapheresis procedure (step 1), implementing on-site CAR-T cell production to reduce transportation/logistics costs and vein to vein time (steps 3 and 9), and exploring alternatives for gene transfer (step 5). These critical steps impair cost and time reduction in the preparation of CAR-T cells, such the expansion time (step 6). In this context, we list potential solutions for streamlining procedures to shorten preparation time and substantially decrease the final product cost, Figure 1.

## Strategies to reduce costs

Despite the proven clinical benefits of CAR-based products, the high cost of producing CAR-T cells to treat each patient remains a significant challenge. The primary contributors to the high cost of CAR-T cell manufacturing are cell culturing, transduction with viral vectors, and the transport of T lymphocytes. Moreover, the current system relies on lentiviral or retroviral vectors, which require the production of large quantities of viruses for clinical-grade use, maintaining a high degree of purity and compliance with GMP standards. This results in high production costs for these vector stocks. Additionally, the use of these vectors necessitates routine testing to detect and exclude products containing viral particles with replication competent retrovirus (RCR). For virus batch release, RCR testing is typically performed on CAR-T cells modified by viral vectors, a test that is not necessary when non-viral vectors are used. We address this challenge by describing potential solutions that, especially when applied together, could help expand the accessibility and affordability of CAR-T cell therapy.

### Non-viral vectors

Strategies that avoid the use of viral vectors can significantly reduce the cost of gene transfer, which is one of the main resources used to prepare the cells. Additionally, certain procedures related to non-virus-based genetic modification can simplify sample and waste handling in CAR-T manufacturing units, thereby reducing operational complexity. With recent advances in genetic engineering, tools such as transposons, CRISPR, nanoparticles, and mRNA can replace viral vectors in the production of CAR-T cells.

Transposons are mobile fragments of DNA that can move within the genome, often referred to as “jumping” genes. These fragments are inserted either randomly or with a biased preference for certain regions of the genome. They operate through a “cut and paste” mechanism and have two main components: the transposon (the DNA fragment to be inserted) and the transposase enzyme, which performs the “cut and paste” function. The transposon genetic unit is flanked by inverted terminal repeat (ITR) sequences, and the transposase enzyme is responsible for recognizing and cutting DNA at the ITR region, subsequently integrating the sequence into the host’s DNA. With this understanding, it has become possible to use these mechanisms to create non-viral DNA modification tools with high efficiency for inserting genes of different sizes.^20^^,^^21^

The main transposon-based systems currently used in clinical trials are Sleeping Beauty (SB) and piggyBac (PB). The SB tool was created using reverse engineering to reactivate the salmonid fish transposase gene and adapt it for use as a genetic tool for mutagenesis and transgenesis in mammalian cells.^22^ PB was identified and isolated more than 30 years ago from the Cabbage Looper genome.^23^ The two vector systems use similar mechanisms consisting of using a transposon and a transposase, which can be delivered to the cell in the form of protein, mRNA, conventional plasmid DNA or minicircle (MC) plasmids. Recently, MCs have been successfully used in CAR-T cell manufacturing with the SB transposon system. MCs are streamlined, non-viral DNA vectors derived from plasmids and synthesized by engineered bacteria expressing an inducible recombinase enzyme. This method results in vectors that carry only the therapeutic transgene under specific promoter control, without the prokaryotic backbone, improving safety and compactness for gene delivery. The MC platform enhances SB transposition and transgene integration, leading to more stable cell modifications, improved CAR-T cell viability, and higher gene transfer rates, all while enabling rapid, virus-free CAR-T cell manufacturing.^24^

This establishes advantages as the delivery mechanism can be carried out by electroporation, for example, eliminating the need of production of viral vectors for efficient delivery of the transgene.^25^ However, there are some differences between the two tools: the SB tool generally integrates into TA-rich sites and has an integration profile that is close to random.^26^ PB, on the other hand, integrates into sites rich in TTAA,^27^ and its integration generally occurs at transcription start sites. Furthermore, PB-based transposon tools can integrate larger transgenes than SB.^20^

The first clinical trial that used SB as a vector to insert CAR into T cells proved the feasibility of this strategy.^28^ In this trial, patients were treated with autologous CAR-T cells (*n* = 7, NCT00968760) or allogeneic cells following bone marrow transplantation (*n* = 19, NCT01497184). In the group that underwent autologous CAR-T cell treatment, the progression-free survival rate and overall survival rate were 83.3% and 100%, respectively, over a period of 30 months. For patients who received allogeneic transplantation, the 12-month progression-free survival rate was 53%, and the overall survival rate was 63%. Patients who received the autologous transplant (and autologous CAR-T cells) were subsequently enrolled in a second clinical study (NCT01492036) for long-term monitoring of the antitumor response achieved by CAR-T cells.^29^ Of the 7 patients, only one had died and four still had detectable CAR-T cells with a median persistence time of 4 years. Another study against B cell hematological malignancies with a new CAR molecule design against CD19 (NCT02807883) is already being carried out and showing positive results regarding tolerability, disease control and cost reduction.^30^ The group of Monza, Italy, has also been successfully using an SB-based CAR expression system in cytokine induced killer cells for treating pediatric B cell precursor leukemias.^31^ Another initiative for SB-based CAR generation, named CARAMBA (NCT04499339), employs CAR-T cells engineered against SLAMF7 for patients with multiple myeloma.^32^ Reports of the results for this trial are expected to be published soon.

PB has also been used to modify T cells in clinical trials, as demonstrated in the recent CARTELL trial, where 10 patients were treated with anti-CD19 CAR T cells.^33^ However, two patients developed lymphoma originating from T cells modified with the PB-based tool. In the analysis conducted, no mechanism was identified that could precisely determine the cause of this transformation of the gene-modified T cells into neoplastic cells, as no consistent alterations in oncogenes or tumor suppressor genes were observed. However, numerous copies of the CAR transgene (an average of 24 copies per cell) were integrated into the DNA of the malignant cells. It is believed that this phenomenon may have triggered dysregulation of cellular homeostasis, leading to tumor development. In other words, the cause may not have been the tool itself. Unfortunately, one patient died because of this adverse event. On the other hand, other clinical trials have shown a good safety profile and efficacy in eliminating tumor cells using PB-based tools. Some of these trials target the CD19 antigen in B cell malignancies^34^ or EGFR in non-small cell lung cancer.^35^

Gene editing-based tools can also be valuable to promote CAR-coding sequencies into the genome of the cell. The main system used nowadays consists of CRISPR-Cas9-based systems. In this system, synthetic guide RNA (RNAg) targeting a specific DNA sequence with high specificity guide a nuclease called Cas9, which promotes a double strand cutting in the DNA. DNA strand breaks are subsequently repaired by processes such as non-homologous end-joining or, when in presence of a donor DNA strand with homologous ends, by the precise homology-directed repair process.^36^

The major advantage of CRISPR-Cas9 editing is its simplicity and efficiency. This tool allows for precise targeting of the DNA region where the CAR can be inserted, resulting in a gain of function for T cells. For instance, inserting the CAR into the constant region of the T cell receptor alpha chain (TRAC) can potentially enhance the potency of CAR-T cells by promoting a tightly regulated pattern of CAR expression.^37^ Additionally, this approach may help mitigate any unintended effects associated with nonspecific recognition through the T cell receptor (TCR). By having this efficient deletion capacity, the Crispr-Cas9 tool can be fundamental to produce universal off the shelf CAR-T cells, which will be described in the topic further.

In addition to editing tools, delivery techniques play a crucial role in reducing costs.^38^ To manufacture CAR-T cells using non-viral methods, electroporation is the most currently used approach. Electroporation releases electrical pulses that promote pores in the cell membrane, causing transient destabilization followed by subsequent membrane integrity restoration.^39^ It is now possible to use automated and closed platforms that can efficiently deliver transport plasmids of different sizes, mRNA and even (ribonucleic) proteins into the cell with low cytotoxicity using electroporation. Electroporation of T cells or PBMC using 4D-Nucleofector System (Lonza),^31^^,^^40^ ExPERT GTx (MaxCyte),^41^ Neon Transfection System (Thermo-Fisher),^33^ and CliniMACS Prodigy (Miltenyi Biotec)^24^ is already validated and prepared for scale-up production. In parallel, nanocarriers^42^ and mRNA^43^ can also be an alternative for the delivery of non-viral systems. The use of mRNA to express CAR-T cells is an intriguing approach due to its transient expression, which can be advantageous in preventing genetic modifications resulting from genomic integration. Additionally, it allows for short-term CAR-T cell-based responses, which may be particularly beneficial when targeting antigens associated to cells involved in autoimmunity, such as CD19 in B lymphocytes. Currently, clinical trials are already underway utilizing mRNA-based anti-BCMA CAR-T cells, with promising results.^44^ Approaches that are using *in vivo* programming of CAR-T cells by nanoparticles have huge potential for reducing treatment time and costs. Some pre-clinical studies have already shown that these particles have the capacity to carry mRNA^45^ or plasmids^46^ and modify circulating T cells *in vivo*.

Therefore, by combining alternative delivery tools such as non-viral vectors, production complexity can be reduced. The use of closed electroporation systems with plasmids, CRISPR, or RNA, for example, can be implemented in laboratories with lower biosafety requirements. This simplification of the dedicated cell manufacturing structure eliminates the need for a complex setup, making it easier to expand CAR-T cell production facilities. This approach could facilitate the establishment of point-of-care units for CAR-T cell manufacturing, reducing the need for patients and cells to travel long distances for cell collection and infusion. The reduced logistics can significantly impact vein-to-vein times and costs associated with cell transportation, enhancing the cost-effectiveness profile of new CAR-T cell therapies. Particularly for newly developed CAR-T cell products that must be validated in first-in-human trials, eliminating the costly and time-consuming step of viral vector production can serve as a fast track to streamline innovations and bring them to patients more quickly in the CAR-T cell field.

### Reduced expansion time

**Figure 2:**
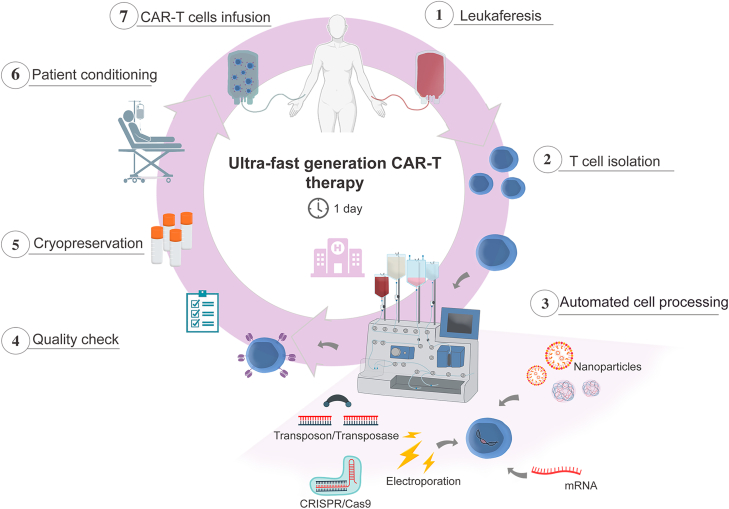
Reduced expansion time of CAR-T cells contributes to the optimization of the production protocol for these cells The automation of the CAR-T cell production process using a closed system can help reduce the production time of the cell product and eliminate the need for transportation to specialized centers. This approach essentially involves removing cells from the patient, modifying them using non-viral methods (such as transposons, nanoparticles, or CRISPR), performing quality control, and reinfusing them into the patient. This process can be completed in just one day.

It is crucial to advance the development of new technologies to improve CAR-T cell therapy, with the aim of both reducing costs and production time. Some strategies for this purpose have already been mentioned, and we emphasize the use of non-viral vectors, such as transposons, due to their simplicity and lower complexity in terms of manipulation. Another critical issue is the time required for CAR-T cell preparation, as shortening the expansion time could be a promising strategy to significantly reduce costs and make the product available in a timely manner for patients who cannot afford to wait (Figure 2).

Currently, research groups have been working to shorten the cell proliferation period of CAR-T cell production. The use of lentiviral vectors as a tool for gene modification has some bottlenecks that preclude ultrafast production of CAR-T cells^47^^,^^48^^,^^49^ that is, there is a requirement for cells to be activated before viral transfection, although the action of lentiviral vectors does not depend strictly on the cell cycle.^50^ One study demonstrated that the efficacy in transducing quiescent T cells to express CAR using lentiviral vectors was low, and when the cells were activated, excellent transduction rates were achieved.^50^ Our group has performed experiments producing CAR-T cells in short protocols (less than 24 h) using transposons systems. Cells prepared this way are effective against human B cell precursor leukemia xenografted into immunodeficient mice. Indeed, the use of transposon-based vectors does not require cells to be in the cell cycle for effective gene-transfer. Besides, we managed to produce functional CAR-T cells in a few hours, without activation and T cell expansion. In a side-by-side experiment of this ultrafast protocol compared to the traditional protocol, in which cells were activated and expanded for 8–12 days, it was possible to obtain comparable survival results.^47^^,^^48^

In the same direction, other groups are also trying to optimize the production of CAR-T cells. In order not to use the classic activation via recognition of CD3 and CD28 for modification using lentiviral vectors, Ghassemi and collaborators previously conditioned T cells with IL-7 and IL-15 or with serum starvation, and after a few hours, CAR insertion was achieved by viral vectors through an overnight incubation, leading to a one-day-based CAR-T cell generation protocol with efficient antitumor activity.^51^ More recently, Novartis has introduced a CAR-T cell development protocol that can now be completed in just three days. Together with a decentralizing CAR-T cell production strategy, this advance results in a significant reduction in the total production and treatment time, reducing the complete period from 30 to 10 days, which includes the transport of cells from the hospital to laboratories specialized in the production of CAR-T cells and their return to the hospital.^52^ The production of CAR-T cells in fully closed and automated systems also allows the production time of these cells to be reduced in addition to reducing the complexity of the manufacturing process.^53^

One aspect under discussion regarding the necessity to reduce the expansion time of these cells concerns the memory phenotype composition of the cell product. T cells can assume different phenotypes, such as Tnaive (Tn), effector memory (Tem), central memory (Tcm), terminal effectors (Tte), and stem cell (Tscm).^54^ Each subtype has important roles in the antitumor activity of CAR-T cells. However, the less differentiated cells, Tscm, have gained prominence in the discussion of the effectiveness of this therapy because these cells might display a high capacity for self-renewal.^55^ In one study, the memory profile of cells was compared during culture, and it was demonstrated that cells that remained in culture for only 3 days had a better ability to control leukemias *in vivo* in a preclinical model.^56^ T cells, when grown for a long period in culture, tend to be mainly composed of Tcm and Tem populations.^57^ On the other hand, once the expansion time is reduced, the proportion of Tscm cells is greater, as the differentiation time is diminished. Therefore, reducing the expansion time of these cells can be advantageous, not only in terms of time but also by creating a less differentiated cell product, potentially leading to reduced doses of CAR-T cells to achieve the same effect. In a phase I study, the CAR-T cells dose infused was 25-fold lower than the dose currently used.^52^

### Off-the-shelf CAR-T cells

Another promising approach to reduce the cost and complexity of CAR-T cell therapy is the use of CAR-T cells generated from healthy donors, known as the off-the-shelf product approach. This method allows the production of CAR-T cells for multiple patients from a single donor. In addition to reducing the cost and delivery time of this cellular product, there is another advantage to using T cells from healthy donors: these donors have not undergone chemotherapy, radiotherapy, or bone marrow transplantation, meaning their T cells are healthier and less compromised in their ability to fight cancer. This is particularly important since many patients have aggressive diseases that prevent them from waiting several weeks to receive CAR-T therapy,^58^ the alternative of a ready-to-use product becomes attractive.

Allogeneic transplantation can induce graft-versus-host disease in patients due to the recognition of recipient’s tissues and cells by donor’s T cells. Therefore, the use of gene editing tools to delete the TCR from CAR-T cells may be an applicable approach to avoid this undesirable side effect.^37^ CRISPR-based tools are suited to this kind of gene editing by using guide RNAs designed to recognize the T cell receptor alpha constant (TRAC) sequence of the TCR.^59^^,^^60^^,^^61^ In addition to deleting the TCR, other genes can be targeted in T lymphocytes to make the cells resistant to specific drugs. One such gene is CD52, which encodes a molecule targeted by cell-depleting antibodies like Alemtuzumab. In a phase I clinical study involving 6 leukemia patients, CRISPR-Cas9 was used to disrupt both the TRAC region and the CD52 gene in T cells. This genetic modification created universal anti-CD19 CAR-T cells, which demonstrated promising results in the treatment of these patients.^60^ In another study, in addition to disrupted CD52 gene and TRAC region to avoid host immune-mediated rejection, a bispecific CAR was added that recognizes CD19 and CD22 molecules.^59^ Using CRISPR to create allogeneic CAR-T cells, it is also possible to create CAR-T cells against tumors originating from T cells. The biggest bottleneck for combating T cell-derived tumors is fratricide, that is, CAR-T cells target each other since both express the protein they’re engineered to recognize. To achieve this, the researchers inactivated 3 genes: TCR, CD52, and CD7 using CRISPR-Cas9. The knockout of TCR and CD52 aims to turn cells into universal CAR-T cells and CD7 is knocked out to avoid CAR recognition.^62^ Some authors estimate that cells prepared from a single healthy donor can be used to treat between 20 and 100 patients.^63^^,^^64^

Another interesting and promising alternative is the generation of CAR-T cells derived from induced pluripotent stem cells (iPSCs). iPSCs are cells that are reprogrammed to return to the embryonic-like pluripotent state with stem-like characteristics. These characteristics become attractive, as once the iPSC-derived CAR lineage is implemented, it is theoretically possible to treat countless patients. NK cells, including iPSC-derived CAR-NK can also be used as an off-the-shelf product as they do not require HLA matching, but it does not generate memory cells.^65^ It is also possible to generate iPSC-derived CAR-T, which can generate memory to improve the persistence of antitumor activity.^66^ Clinical studies with iPSC derived NK or CAR-T positive cells, are already in phase I clinical evaluation.^67^ Allogeneic CAR-T cells are thought to persist for shorter periods in patients compared to autologous cells. While this may limit long-term antitumor control or even prevent complete tumor elimination, in the context of autoimmune diseases, where CD19-targeted CAR-T cells are used to eliminate potentially autoreactive B cells, long-term persistence may not be necessary. In these cases, short-term persistence could be sufficient to reset the B cell compartment and eliminate the autoreactive repertoire. However, this assumption still requires validation through clinical practice. Therefore, the generation of off-the-shelf CAR-T cells represents a promising alternative for providing therapy to a larger number of patients.

**Figure 3:**
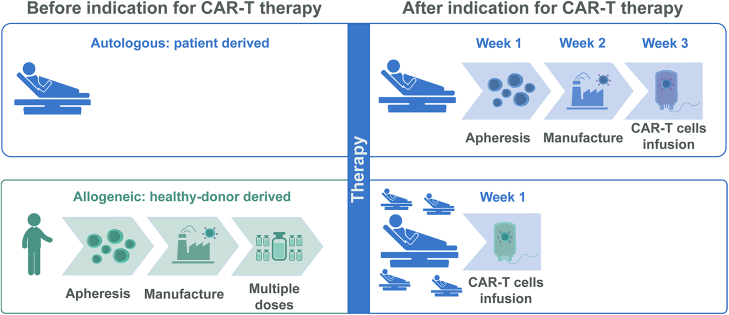
Off-the-shelf CAR-T cells offer an alternative to reduce both the time and cost for patients to receive treatment Currently, most CAR-T cell therapies are autologous, involving the extraction, modification, and reinfusion of a patient’s own cells—a process that can take up to three weeks. An alternative to streamline this process is to utilize universal CAR-T cells derived from allogeneic donors, producing multiple doses to treat several patients in less than a week. This approach is referred to as off-the-shelf CAR-T cell therapy. In addition to reducing time and cost, donor cells may be healthier, as they have not been exposed to the invasive treatments that cancer patients typically undergo, such as chemotherapy and radiotherapy.

This approach could significantly reduce the cost of manufacturing cellular products. Additionally, the time required for patients to receive treatment would be greatly shortened, as they would no longer need to wait for their own cells to be manufactured (Figure 3).

### Decentralizing manufacture

Currently, more than 200 clinical trials in the US are focused on optimizing the CAR-T cell therapy process. These trials aim to enhance efficacy, reduce toxicity, and broaden applications to include solid tumors, autoimmune diseases, and infectious diseases. One major challenge is related to the centralized manufacturing approach that leads to limited CAR-T cell production capacity, long time for products preparation, complex logistics, and elevated cost within the pharmaceutical industry.^68^

Patients often face delays of up to three weeks as they await a denominated “manufacturing slot”, during which the pharmaceutical company can initiate the production of CAR-T cells using the patient’s autologous apheresis product. For patients enrolled in clinical trials with aggressive diseases, this waiting period, averaging 3 to 4 weeks, is often impractical. In many countries, leukapheresis products (fresh or frozen) are sent to the United States or European Union for manufacturing. Once ready, it is frozen and shipped back, resulting in a process that can take over a month and demands very complex logistics, cold, and custody chain.^69^

CAR-T cell manufacturing typically follows a centralized, standardized protocol overseen by major pharmaceutical entities. However, decentralization efforts, led by academic research centers and university-affiliated institutions, represent an alternative. In these settings, smaller-scale manufacturing using open systems occurs, requiring advanced technical and structural capabilities. This decentralized approach poses challenges in meeting stringent quality control and efficacy requirements. An efficient alternative to solve this involves deploying closed, automated systems, as described earlier, covering the entire process from apheresis to CAR-T cell expansion.^70^

This approach streamlines manipulation steps, enhances reproducibility, and eliminates the need for a tightly controlled environment through standardized, pre-established kits. The point-of-care (POC) approach in CAR-T cell generation decentralizes manufacturing to medical-hospital care facilities, reducing risks associated with product loss and transportation costs. POC production not only introduces the possibility of administering the product freshly to patients but also significantly reduces costs and production time.^71^ Indeed, technological advancements, such as advanced bioreactors and automated closed systems, are making POC production more accessible by eliminating the need for costly cleanrooms meeting high standards.^68^

The main POC manufacturing platforms include the CliniMACS Prodigy (Miltenyi Biotec), Cocoon (Lonza) platforms, and G-Rex (Wilson Wolf). The Cocoon is a closed manufacturing system based on a single-use transportable cassette that internalizes all media and reagents and maintains the components in a temperature-controlled environment, although T cell enrichment takes place in a separate platform prior to manufacture start is required. The CliniMACS Prodigy is a closed system that enables the enrichment and depletion of specific cell types using magnetically labeled antibodies. The CliniMACS Prodigy system automates all necessary steps of CAR-T production beyond T cell enrichment, such as activation, transduction, washing, and media addiction, in one closed tubing system. An electroporation attachment is now available, allowing for gene editing capabilities and for the use of CAR-T cell protocols based on transposable-elements. The advantage of this platform is that it ensures GMP compliance and reduces strict cleanroom requirements. Wilson Wolf’s G-Rex technology built on a gas-permeable membrane with enables high cell density production over a short culture time, at a lower cost compared to fully automated systems. Additionally, closed system G-Rex bioreactors with validated sterile fluid paths are available to simplify and accelerate the concentration of the cell product and cell harvesting, utilizing a specialized positive air pressure pump, the GatheRex.^72^^,^^73^

Lastly, adopting non-viral vectors for genetic modification simplifies the CAR-T cell manufacturing reagents, as opposed to viral vectors that require complex and costly structures to be produced. This holistic view emphasizes the importance of optimizing manufacturing processes to ensure timely and cost-effective delivery of CAR-T cell therapies. An alternative to viral vectors in face to modify T lymphocytes is by electroporation using transposon-based non-viral vectors. CliniMACS Prodigy Electroporator into the Prodigy platform, the Cocoon platform with the 4D-Nucleofector (Lonza), and the GTx Electroporator from MaxCyte integrated automate the movement of cells through electroporation, reducing hands-on time for this objective. These systems can transfect up to 20 billion cells in current good manufacturig practice (cGMP) compliant cartridges with an established regulatory pathway supported by regulatory agencies.^74^

### Process improvement

The success of CAR-T cell therapy, particularly in the treatment of B cell leukemias and lymphomas, underscores the urgency of refining the development of an even more effective and functional final product. The standard protocol for CAR-T cell generation, although with slight variations depending on the product, generally involves obtaining leukocytes, enriching T lymphocytes, activating T cells, and then expanding the cells. Hours to days after T cell activation, these cells are transduced using viral vectors to express the CAR transgene.

Among the main culture media used in the manufacturing of available products are RPMI-1640 (Gibco), AIM-V (Thermo), Optimizer (Gibco), TexMACs (Miltenyl), and X-VIVO (Lonza). In a comparative study between RPMI-1640, AIM-V, and Optimizer, all supplemented with autologous serum and in the presence of IL-2 (175 IU/mL), it was demonstrated that, under these specific conditions, RPMI showed better performance, with the result being dependent on the serum concentration in the culture medium.^7^

Serum-free media have also been evaluated, as the impact of serum use reflects both on efficiency and on quality control and safety for regulatory purposes. In this scenario, another comparative study using serum-free media Optimizer, X-VIVO, and TexMACs showed that Optimizer had better performance in terms of the total number of CAR cells at the end of the expansion period when cells were stimulated with OKT-3 in the presence of IL-2 (300 IU/mL) for 6 days.^75^^,^^76^^,^^77^

In many current protocols, CAR-T cells are expanded in the presence of IL-2, including commercial products, such as KYMRIAH and YESCARTA. The cytokine IL-2 is related to various functions in T lymphocytes, mainly in the differentiation and proliferation of these cells. IL-2 can lead to differentiation of T cell phenotypes into Th1, Th2, and Treg profiles, depending on its concentration and exposure time. Several preclinical studies reported the use of other common gamma-chain (γc) cytokines such as IL-7, IL-15, and IL-21 showing more effective T cells when using different combinations including IL-7/IL-15 and IL-15/IL-21.^78^^,^^79^

CAR-T cells cultured *ex vivo* and supplemented with IL-2 exhibited the least anti-tumor effect compared to the combination of IL-15 and IL-21. Furthermore, IL-15/IL-21 demonstrated an increased capacity to enhance CAR-T tumor killing. Additionally, the same research group demonstrated that IL-7 and IL-15 promote the *ex vivo* expansion of CAR-T cells, whereas IL-15 and IL-21 appear to be better suited for *in vivo* administration following CAR-T cell infusion.^80^^,^^81^ Another promising strategy includes the modification of CAR-T cells to produce IL-18, which has demonstrated enhanced antitumor capacity and efficacy in both animal models and cell cultures.^82^^,^^83^^,^^84^

### Patient’s biomarkers to enhance CAR T cell treatment efficacy

Studies based on new biomarkers on patients under CAR-T therapy have been crucial for understanding patterns associated with CAR-T cells, and tumor cell characteristics, as well as markers of response resistance. By investigating the expression of target antigens, pre-treatment immune profile, tumor characteristics, and cytokine profile, these research endeavors aim to identify predictive biomarkers of treatment response, as well as factors associated with toxicity and therapeutic efficacy.

Despite the success of CAR-T cell therapy, some patients experience primary resistance (PR) to treatment or relapse after an initial response. The resistance mechanisms observed in CD19-specific CAR-T cell therapies can be broadly divided into two main categories: target antigen-positive and target antigen-negative tumor cells. Primary resistance to CAR-T cells occurs in 10–20% of pediatric patients with B-ALL and approximately 30% of CD19^+^ lymphomas. For instance, a high tumor burden at the time of lymphodepletion has been associated with CAR-T cell therapy failure in ALL and lymphoma.^85^^,^^86^

The prognosis is particularly poor in patients with chronic lymphocytic leukemia (CLL): the 18-month progression-free survival (PFS) rate was 29% with tisagenlecleucel (tisa-cel).^87^ Among the key factors, the functional state of the patient’s immune system emerges as a significant issue associated with the diminished anti-tumoral efficacy of CAR-T cells, particularly in individuals with compromised T cell populations, as observed notably in patients with CLL. In such instances, the presence of deficient T cells poses challenges during the autologous product preparation, with the exhaustion phenotype displayed by T cells likely serving as a causative factor in CAR-T cell dysfunction. Indeed, phenotypically exhausted T cells are characterized by elevated levels of inhibitory receptors, reduced cytotoxicity, and diminished proliferative potential.^88^

An interesting study conducted by Mackall and her colleagues involving two cohorts of patients with large B cell lymphoma treated with axi-cel evaluated the association between the phenotypic profile of CAR-T cells infused in these patients and the clinical progression of the disease. The authors observed that CAR T cells recovered from the bloodstream in some individuals, 7 and 21 days after infusion, exhibited a CAR-TReg (regulatory T cell) phenotype. The CAR-TReg phenotype presence in treated patients associated with LDH levels showed as a potential biomarker for poor prognosis after treatment in these individuals.^89^

In a recent prospective transcriptomic study, myeloid cells emerged as biomarkers in response and survival among patients with relapsed or refractory large B cell lymphoma (LBCL R/R) undergoing anti-CAR-T CD19 therapy, particularly with tisagenlecleucel (tisa-cel) and axicabtagene ciloleucel (axa-cel) products. A four gene signature associated with myeloid cells, expressed by isolated T cells from leukapheresis products, correlated with notably short progression-free survival, emphasizing monocyte impact on CAR-T therapy response. Consequently, elevated absolute counts of circulating monocytes post-allogeneic CAR-T therapy adversely affected response and progression-free survival in high-risk LBCL R/R patients.^90^

Additionally, another study demonstrated that the therapeutic effectiveness of CAR-T cells correlates with the cytokine and chemokine production profile in each treated patient. Moreover, older patients exhibit a poorer prognosis following therapy compared to younger patient cohorts, thus highlighting age as a significant risk factor. Other resistances mechanisms inherent to the tumor itself have become a pivotal focus in evaluating factors associated with refining CAR-T cell therapy strategies. Tumor survival factors such as Fas ligand (FasL) or cytokines like IFN-gamma are directly correlated with tumor-free progression in treated patients.^91^

Furthermore, aside from these markers, Sing and colleagues have described that pro-apoptotic molecules such as FADD, BID, CASP8, or TNFRG10 are implicated in instances of relapse post-therapy.^91^ Additionally, programmed death-1 ligand-1 (PD-L1) expressed by the tumor cells or the tumor microenvironment inhibits CAR-T cell cytotoxicity in B cell malignancies and have been associated with poor immunotherapy success.^92^^,^^93^

A recent and pioneering study conducted by Khan and colleagues investigated the epigenetic profiling of DNA hypermethylation at genes regulated by a complex called PRC2 in embryonic stem cells. Employing a cohort of 14 patients, they demonstrated a decrease in accessibility at these PRC2 target genes, coupled with an increase in accessibility of regions characteristic of hematopoietic stem cells and progenitor’s lineage, particularly in individuals who exhibited non-responsive outcomes to CAR-T cell therapy. The study focused on leukemic subpopulations expressing multilineage markers and revealed a diminished antigen presentation in non-responder patients. These findings suggest a correlation between epigenomics mechanisms and resistance to CD19-CAR therapy.^94^ A recent publication demonstrates that interfering with PRC2 in CAR-T cells during *in vitro* culture by using epigenetic probes can enhance antitumor functions.^95^

Presently, this multidimensional approach provides crucial insights to optimize patient clinical management, driving significant advances in CAR-T therapy providing new tools for understanding patients eligible for treatment. These studies help to define criteria for patients who are most likely to respond to CAR-T cell therapy and try to design new therapeutic approaches for those patients who possibly will not respond.

## Conclusion

CAR-T cell therapy has revolutionized cancer treatment, demonstrating excellent efficacy in patients with hematological tumors. However, the process of producing these cells is complex and involves several steps that increase the cost of this therapy, limiting access for patients who need it. Efforts are already being made to bring this therapy to the broader population. There are several aspects of the production process that should be optimized to minimize costs, such as the use of non-viral vectors, protocols that do not require cell expansion, decentralization of manufacturing, improvements in the cellular product generated, and a better understanding of patient and CAR-T cell characteristics associated with successful treatment outcomes. Therefore, further research and optimization of the manufacturing protocol are still needed.

The primary objective of this review is to promote the dissemination of potential strategies aimed at reducing the cost of this highly effective therapy. While healthcare is a fundamental right of all individuals, access to it remains unequal, particularly in low- and middle-income countries. The development of new therapies is a highly complex process, requiring significant effort, and financial investment. Nevertheless, scientific advancements over the years have demonstrated alternative approaches that could expand access to treatment for a broader population. It is therefore essential to intensify investments in these initiatives with greater commitment. Although efforts are already underway in countries, such as Brazil, India, and South Africa,^96^ significant challenges persist in achieving widespread and equitable access. Luckily, some of the strategies discussed here may contribute to overcome these barriers.

## Acknowledgments

The authors receive scholarships and financial support from the 10.13039/501100006506Brazilian Ministry of Health and the funding agencies Carlos Chagas Filho Foundation for Research Support in the State of Rio de Janeiro (10.13039/501100004586FAPERJ) and the 10.13039/501100003593National Council for Scientific and Technological Development (CNPq). The authors are thankful to the company Shapes Esquemas Didáticos for providing the figures.

## Author contributions

The core concept of the theme was developed by L.A. The design, writing, and research were carried out by L.A. and L.R.B.-S., with revision and support provided by M.H.B.

## Declaration of interests

The authors declare no competing interests.
